# The Social Modulation of Imitation Fidelity in School-Age Children

**DOI:** 10.1371/journal.pone.0086127

**Published:** 2014-01-22

**Authors:** Lauren E. Marsh, Danielle Ropar, Antonia F. de C. Hamilton

**Affiliations:** 1 School of Psychology, University of Nottingham, Nottingham, Nottinghamshire, United Kingdom; 2 School of Psychology, University of Surrey, Guildford, Surrey, United Kingdom; 3 Institute of Cognitive Neuroscience, University College London, London, United Kingdom; Birkbeck, University of London, United Kingdom

## Abstract

Children copy the actions of others with high fidelity, even when they are not causally relevant. This copying of visibly unnecessary actions is termed overimitation. Many competing theories propose mechanisms for overimitation behaviour. The present study examines these theories by studying the social factors that lead children to overimitate actions. Ninety-four children aged 5- to 8-years each completed five trials of an overimitation task. Each trial provided the opportunity to overimitate an action on familiar objects with minimal causal reasoning demands. Social cues (live or video demonstration) and eye contact from the demonstrator were manipulated. After the imitation, children's ratings of action rationality were collected. Substantial overimitation was seen which increased with age. In older children, overimitation was higher when watching a live demonstrator and when eye contact was absent. Actions rated as irrational were more likely to be imitated than those rated as rational. Children overimitated actions on familiar objects even when they rated those actions as irrational, suggesting that failure of causal reasoning cannot be driving overimitation. Our data support social explanations of overimitation and show that the influence of social factors increases with age over the 5- to 8-year-old age range.

## Introduction

Observation and imitation of other people's actions is an important way for children to learn about the world, reducing the need for costly trial-and-error [1] and see [2] for a review). However, learning by observation is complicated by the fact that some objects are not transparent in their mechanism [3] and on some occasions the demonstrator may behave inefficiently due to habit, error, or lack of experience with the object. In order to efficiently interact with an object, the child must filter out actions that are causally necessary from those that do not contribute to the completion of the action [4]. Failure to exclude unnecessary actions from a sequence is termed overimitation [5], whereas efficient pursuit of a goal alone is termed goal emulation [6]. In this paper we investigate which cues influence children's imitation fidelity and how these cues change with development.

Horner and Whiten [7] demonstrated that chimpanzees are remarkably good at goal emulation if information about action causality is available. However, in the same task, a group of 3- to 4-year-old children failed to emulate the goal of the action, instead choosing to faithfully imitate both the necessary and unnecessary actions demonstrated. Many subsequent studies have demonstrated that from around 14 months of age, children overimitate actions, despite visible evidence that they are not causally relevant [5], [8]–[11].

There are multiple theories which attempt to explain overimitation in children. Broadly speaking, these theories fall into causal reasoning explanations and social explanations, although nuances within these categories are debated. Firstly, causal reasoning explanations follow the argument that if a demonstrated action upon an object is perceived as intentional, the child will believe that the action is important. This judgement of importance may be due to the demonstration distorting the child's causal understanding of the object such that they believe the action is necessary to achieve the goal (automatic causal encoding hypothesis, [5]) or it may be judged as important but that the purpose of the action is unknown (unspecified purpose hypothesis, [7], [11]). In this case, it is a ‘safe bet’ to copy everything and to refine later [11]. Alternatively, social hypotheses propose that overimitation performs a social function. Either the child has a desire to be like the demonstrator and finds it intrinsically rewarding to share their experiences (shared experience hypothesis, [12], [13]) or they want to communicate ‘likeness’ with the demonstrator in an attempt to affiliate (affiliation hypothesis, [14]). Finally, a recent theory proposes that children learn prescriptive norms about the ‘right way’ to do things when actions are demonstrated. Therefore overimitation occurs because children are conforming to these perceived norms (normative behaviour hypothesis, [15], [16]).

There is very little consensus over which of these processes may be driving overimitation; some may work in combination, or at different ages. One previous review paper argues that all of these hypotheses can explain some part of overimitation behaviour by emphasising the role of the child's goal in an imitative situation [17]. This theory can also explain the co-occurrence of selective imitation and overimitation in children. Over and Carpenter propose that a child can adopt a social goal, a learning goal or a learn-to-be-social goal in an imitative situation and this goal determines whether they faithfully or selectively imitate. With a social goal, their priority is to imitate the model faithfully as this serves an affiliative function, similar to mimicry. Evidence for this account comes from studies showing more overimitation when a child interacted with a sociable demonstrator compared to a demonstrator who was socially aloof [13], and when the demonstration was presented live rather than in a video [10], [18]. Additionally, children primed by observing ostracism were more likely to overimitate than children who witnessed a comparable scenario without ostracism [14]. In contrast, under a learning goal children are more likely to be selective in their imitation. This is evidenced by a study which demonstrates that children given a social copying task prior to an overimitation task were more likely to overimitate than those children given a collaborative learning task first [19]. Here, the child's goal is changed by the aims of the initial task. Finally, Over and Carpenter [17] propose that with a learning-to-be-social goal, the child aims to learn the social rules of a given situation. This view parallels the normative behaviour hypothesis and explains why children justify their overimitation in terms of normative language [15] and protest when someone else omits the unnecessary action [16]. While the theory proposed by Over and Carpenter [17] has good explanatory power and pulls together a diverse range of theories, it is not yet clear how children adopt a specific goal and what cues they use to switch between them.

One potential reason for the diversity in explanations of overimitation behaviour may be because the field is muddied by the diverse range of overimitation tasks and the precise definition of overimitation. Originally, overimitation was classed as the imitation of *visibly* irrelevant actions [7]. However, the objects used in demonstrations vary considerably with respect to how mechanically complex they are (complex: [9] vs simple: [20]) and whether they are transparent [9], [10], [21] or opaque [12], [22]. Many studies make the assumption that because an object is physically transparent, the actions performed upon it are cognitively transparent to children, however a recent study demonstrates that children make errors in ascribing action relevance for even very simple objects [15]. Therefore, children's replication of actions on these more complex objects may be due to object learning, rather than social drives [20]. This can explain why causal reasoning explanations have been provided for studies involving overimitation on mechanically complex objects [9]. Recent studies have also extended the definition of overimitation to include faithful imitation of tool selection [14] and tool use (when it is simpler to use your hand, [18]) or imitation of the number of irrelevant actions performed [23]. These can be considered faithful reproductions of action but may be functionally different to classic overimitation in which a causally irrelevant action is completed. To ensure that children understand the mechanics of how each object works, and therefore ensure that the irrelevant action is visibly so, the present study utilises very simple, transparent objects that have no mechanical components and do not involve the use of tools to operate. Furthermore, irrelevant actions on the objects are hand actions that do not result in physical outcomes (noises or changes to the appearance of the object). This should prevent object-learning based imitation being coded as overimitation.

In the present study, we test how overimitation is modulated by social cues, causal reasoning and a child's age. First, we suggest that children will adopt a social goal and overimitate for social reasons if the learning component of the task is reduced. To test this, we compare rates of overimitation for live and video demonstrations using simple, non-mechanical objects. Previous studies suggest children copy more when seeing a live demonstration compared to a video demonstration [10], and this increase in overimitation persists when the demonstration is given via a live video feed compared to a pre-recorded video [18]. These data suggest that the opportunity for social interaction drives increased overimitation rather than the reduced quality of the video leading to a performance deficit [24]. We predict that in our task, children will overimitate despite the objects being causally transparent and that overimitation will increase for the live demonstration condition.

We also test the role of another social cue – eye contact – in influencing imitation behaviour. Eye contact is a powerful ostensive cue which may signal communicative intent to an observer [25], [26]. Within the context of a social learning task, direct eye contact may highlight a particular action as relevant or important to the observer in a way that promotes teaching and learning [25], [27]. Therefore, we should expect direct eye contact to increase overimitation when a task requires object learning. Indeed, Brugger et al [4] showed that 14-to 16-month-olds overimitate following social engagement (eye contact and a relevant comment) more than a non-engagement condition (look to wall and an irrelevant comment). However, the study by Brugger et al. [4] does not distinguish between the verbal and eye contact cues. Further, these cues were presented at the start of each action sequence and so we cannot say whether the cue is increasing general attention to the demonstration or whether it is specifically prompting the infant to complete the unnecessary action. Finally, a recent study presents contrasting results suggesting that eye contact may reduce imitation in young children [28]. The present study investigates the role of eye contact in an overimitation paradigm further by examining whether eye contact modulates overimitation when it precedes a necessary or an unnecessary action. In addition, we assess the effect of eye contact on overimitation for familiar objects.

The manipulation of eye contact also allows us to examine the relationship between overimitation and mimicry. Mimicry is the spontaneous copying of actions which have no goal. Some theories suggest that social overimitation and mimicry are functionally related [14], because they are modulated by the same social conditions. For example, overimitation and mimicry both increase when interacting with people with high social status compared to low social status [21], [29], [30], and both decrease following exposure to ostracism cues [14], [31], [32]. We also know that eye contact enhances mimicry [33], which implies that it should enhance overimitation too. Thus, our eye contact manipulation will provide a test of whether mimicry and overimitation are modulated in the same way for this type of social cue.

Another puzzling feature to emerge from studies of overimitation is that this behaviour actually increases throughout early childhood (ages 2- to 5-year-olds [10], [34], and remains in adulthood [34], [35]. One previous study has looked at overimitation in children across a wider age range (2-to 13-year-olds) in Kalahari Bushmen children [22]. Again, an increase in overimitation is reported in this sample yet the authors do not discuss the implications of this finding. We hypothesise that an increase in overimitation may be due to increasing sensitivity to social cues throughout development. As children start school and form new friendships outside their family group, their social skills are likely to improve and their propensity to overimitate may also increase. If there is a relationship between overimitation and social skill development, this relationship should emerge most clearly within the period that these social skills develop. The present study will systematically test how imitation fidelity changes over the 5- to 8- year age range and assess whether these changes are related to changing sensitivity to social cues.

A further way in which to test whether overimitation is socially motivated is to ask participants to explicitly rate the actions as necessary or unnecessary. In a previous study, 5-year-old children were asked to report whether they will perform the unnecessary action and to justify their decision prior to acting [15]. While only 10% of participants justified the unnecessary action as causally relevant, the remaining 90% were unable to justify why they would complete the unnecessary action. However, a caveat of this study is that children were only included in the analysis if they completed the unnecessary actions. It is interesting to study the differences in ratings between children who choose to faithfully imitate and those who do not as this may provide insight into their decision-making process.

Overall, the present study will test four hypotheses. Firstly, if overimitation is socially modulated then overimitation will occur more in situations that elicit more social engagement. Thus, unnecessary actions that are demonstrated live will be overimitated more frequently than those demonstrated in a video. The second hypothesis examines the role of eye contact in overimitation. If overimitation and mimicry are operating on the same social mechanisms [17], then eye contact prior to an unnecessary action should increase overimitation of that action, as it does for mimicry [33]. Third, this study will investigate the developmental change in overimitation. Previous studies have reported overimitation in children aged between 14-months and 13-years or in adults but no study has tried to link developmental changes in overimitation behaviour to the development of other social and cognitive processes. Finally, no previous study has linked overimitation behaviour to explicit ratings of the rationality of the demonstrated actions. If children overimitate for causal reasons, they should report all actions as sensible whereas if they are socially overimitating, they should report the unnecessary action as silly.

## Method

### Participants

All parents gave written, informed consent and the study was approved by the University of Nottingham Ethics committee. Ninety-four children aged 5- to 8-years took part in this experiment. The final sample consisted of 26 five-year olds (16 female), 25 six-year-olds (8 female), 22 seven-year-olds (12 female) and 21 eight-year-olds (11 female). Children were recruited through the ‘summer scientists week’ scheme at the University of Nottingham. Groups of children from the local area were invited to come and take part in a fun session of games and experiments during their summer holidays.

### Design

A mixed model design was used. Children were randomly assigned to one of two between-participant experimental conditions (live demonstration or video demonstration) that were matched for age and gender. Eye contact was manipulated within participants. Eye contact was counterbalanced for action (either preceding a rational or irrational action) and for trial (which apparatus was used) across participants.

### Stimuli

The action sequences used in the practice and experimental trials are detailed in Table 1. Movies were created for each trial of the video demonstration condition. These commenced with a demonstrator (D) sitting at a table with the trial apparatus in front of her. Over the course of the movie, D performed the sequence of actions required to complete the goal (see Table 1). Within each trial D once paused and looked at the camera for approximately 1 second before looking down and continuing the action. The eye contact either directly preceded a rational action or directly preceded an irrational action. Thus, two versions of each demonstration were filmed. For example, when building the block tower, version one shows D place block one in the centre of the table, pause and look directly at the camera, then continue by picking up block two, rotating it 360 degrees before placing it on block one (in this case, eye contact occurred directly prior to the irrational action) and finally placing block three on top of block two. Version two shows exactly the same action sequence but the pause and eye contact occurred before picking up block one (directly prior to a rational action).

**Table 1:** Descriptions of each action within each trial.

Goal	Action 1	Action 2	Action 3
**Practice trials**			
Make a pattern with beads on the rack	Place bead 1 onto a peg	Place bead 2 on top of bead 1	Place bead 3 on top of bead 2
Put doll into a container	Remove lid from container	Put doll into the container	Put lid back on container
**Experimental trials**			
Retrieve toy duck from box 1	Unclip fastenings of box (R)	Tap the top of the box twice with index finger (I)	Remove the lid of the box and retrieve duck (R)
Retrieve toy elephant from box 2	Remove elastic band (R)	Slide box along the table and back again (I)	Remove the lid of the box and retrieve elephant (R)
Retrieve toy lion from box 3	Pull box towards you (R)	Stroke the top of the box twice with index finger (I)	Remove the end of the box and retrieve lion (R)
Build tower of blocks	Place block 1 in centre of table (R)	Turn block 2 360° (I)	Place block 2 on top of block 1 and place block 3 on top of block 2 (R)
Make a paper fan	Gather up concertina paper (R)	Tap paper on the table twice (I)	Fold the paper in half to produce a fan (R)

(R) indicates a rational action. (I) indicates an irrational action.

### Procedure

For testing, each child sat at a child-size table next to the experimenter (E). Parents were present if the child preferred it, and sat behind the child so that they were not distracting. A video camera recorded the child's actions to allow independent coding of imitation behaviour.

All children started the experiment by completing two practice trials. Practice trials were included to ensure that the participants were able to meet the basic motor demands of the task. They also familiarised participants with the routine of the study – first they watched an adult playing with some toys, then they will be given the opportunity to play themselves. During piloting this was found to reduce the child's attempts to reach out for the objects before the demonstration. In practice trial 1, E said *‘I am going to make a pattern with some beads on this peg. When I am done, I would like you to make the same pattern on a different peg so watch me carefully’*. E then placed three beads, one at a time, onto a peg. She then offered the three remaining beads to the child and said *‘Now it is your turn, can you make a pattern on this peg’* (pointing to a different peg). Praise was given on completion, regardless of whether the same or a different pattern was made. For practice trial 2, E said *‘Next up, we are going to play with my doll called Ted. He wants to hide in the pot. Watch me carefully and then you will get a turn to hide Ted.’* E then takes the lid off the pot, places Ted in and puts the lid back on. When finished, E then takes Ted out of the pot, hands him to the child and says *‘Now it is your turn, can you hide Ted in the pot?’* Upon completion, E praised the child. All the children were able to complete both of the practice trials without difficulty.

After the practice trials, the experimental trials began. E said *‘Now we are going to play with some more toys but this time you can see Kate playing first. Let's see what toys Kate has.’* For children assigned to the video demonstration condition, E produced a laptop and placed it in front of the child. E then ran a matlab script which presented the trials in a random order. Each trial started with E saying *‘Look Kate has a toy [duck]’* whilst showing the child a picture of the last frame of the movie that depicted the end goal of the action. E then continued by saying *‘Kate is going to show you how she got the [duck] out of the box’, watch her carefully and then you will get a turn.’* E then played the movie demonstration. Once the movie was over, E put the laptop to one side (still displaying the end goal on the screen) and gave the child the apparatus whilst saying ‘*Can you get the [duck] out’, do it as quickly as you can’*. The name in square brackets was substituted on each trial for the name of the object to be retrieved or built. Note here that the instructions emphasise the action goal and speed, but not the means by which the action is achieved. This instruction ensures that children clearly understand their goal in the situation and should reduce any copying that is driven by demand characteristics. These instructions have been used previously and rates of overimitation are comparable to studies with other instructions [20]. The child attempted the task, was praised and then started a new trial.

Once all five trials were completed, the children were then shown 10 short clips from the action sequences again. Five of these were rational actions and 5 were irrational, presented in a random order. After the clip, they were given a 5-point scale with a smartly dressed man above the 1 and a clown above the 5. They were asked how sensible (E points to the smartly dressed man) or how silly (E points to the clown) was that action? E noted down the child's verbal or point response and moved on to the next clip.

The procedure for children assigned to the live demonstration condition was the same as for the video demonstration except there was no laptop. E had laminated photographs of the goal of the action to put in front of the child. Trial order was randomised by shuffling the photos between each participant. E's script was identical to the video condition. When it was time for the demonstration, a demonstrator (D) brought the apparatus to the table and sat directly opposite the child. When cued by E, D performed the action sequence. Then D reset the stimulus objects to their original configuration behind a screen, then removed the screen and moved out of sight. E then handed the object to the child, with the same instructions as the video condition. Throughout the live demonstrations, a second video camera was positioned to capture D's actions in order to check that the live demonstration was accurate. After all five trials, D came back and performed the same 10 sections of the action sequences that were used in the video ratings. After each, the child was presented with the sensible/silly action rating scale and was asked by E to rate the action.

In addition to the overimitation task, each child completed the British Picture Vocabulary Scale II (BPVS-II), a standard measure of verbal abilities [36] with a separate researcher and parents completed the Social Aptitudes Scale (SAS, [37]), a general measure of social abilities. These measures were completed to check that participants in the live and video groups did not differ on verbal ability or social skills and were entered as predictors in a regression model to predict overimitation.

### Coding and Data Analysis

All coding was based on the video recordings. The coder was blind to the eye contact condition whilst coding the movies. However, the coder was able to tell whether the child had received live or video demonstrations based on the experimental setup. For each trial, the coder was asked to judge whether the goal of the action was achieved and whether the irrational action was performed by the child. The irrational action was judged to be performed if the child made a definite and purposeful movement on the object, as described in Table 1. For each trial, the child was awarded a score of 1 for each irrational action completed and 0 otherwise. Therefore, each child had a total score out of 5 for overimitation. Data from 35 children (37%) were double coded by an independent coder. Overall agreement between coders was 93%. Cohen's Kappa  = 0.92.

All children were able to achieve the goal of each action so this was not analysed. There were no significant gender differences or gender by overimitation interactions within this dataset so gender shall not be considered further. Table 2 shows participant statistics for each randomly-allocated group. As there was a group difference for BPVS and SAS scores (see Table 2), all analyses include these scores as covariates to partial out the variance attributed them.

**Table 2:** Participant group characteristics.

	Live	Video	Difference (p)
N	42	52	
Age	6y9m (1y1m)	6y8m(1y1m)	0.67
BPVS	109.9 (10.6)	115.6 (10.2)	0.01
SAS	26.2 (5.1)	23.3 (5.6)	0.01
Overimitation	2.9 (1.9)	1.2 (1.5)	0.001

Numbers displayed are group means (and standard deviations) for participants in each condition.

For data analysis, we ran three ANCOVAs to test each of our three main research questions (every model included age as a factor), followed by a logistic regression incorporating all variables. First, we compared the effect of live and video demonstration. The total overimitation score out of five for each child was entered into a univariate ANCOVA, with demonstration type entered as a between-participant factor and age in years and months, SAS and BPVS entered as covariates.

Second, the effects of eye contact on overimitation were analysed on a trial by trial basis, due to an unequal number of trials with and without eye contact per child (either two or three). Thus, demonstration type, direct eye contact preceding an irrational action and apparatus type were entered into a univariate ANCOVA as between trial factors and age, BPVS and SAS were added as covariates. Interaction terms for demonstration type and eye contact with age and demonstration type with eye contact were also entered into the model.

Third, rationality ratings were analysed by calculating a rationality difference score for each trial, by subtracting the child's rating of the rational action from their rating of the irrational action. Thus each trial for each child had a rationality rating ranging from −4 (irrational actions rated as more rational than rational actions) to 4 (irrational actions rated as more irrational than rational actions). A score of 0 reflected no perceived difference in rationality between the rational and irrational action. We tested if overimitation on a trial is related to the later rationality rating given on that trial. Rationality difference scores were also analysed on a trial-by-trial basis and entered as the dependant variable into a univariate ANCOVA. Overimitation behaviour, eye-contact and demonstration type were entered as a between trial factors and age, BPVS and SAS were entered as covariates.

Finally, we performed a binary logistic regression to establish which factors are good predictors of overimitation behaviour. Age, BVPS, SAS, demonstration type, eye contact and rationality ratings were entered as single variables and demonstrator eye contact by age, demonstrator eye contact by condition and rationality ratings by age were entered as interaction terms. All variables were entered into a backwards likelihood ratio model.

## Results

Sixty-two percent of children completed at least one unnecessary action in at least one trial in this sample. Rates of overimitation, split by demonstration condition and apparatus type are presented in Table 3. Participants in the live demonstration condition consistently overimitated more compared to those in the video demonstration condition. Overimitation behaviour also differed by apparatus type as participants overimitated least on the fan trial compared to all other apparatus types. Apparatus type was therefore modelled as a nuisance variable in all analyses and will not be considered further.

**Table 3:** Percentage of trials in which overimitation occurred, split by demonstration and apparatus type.

Trial Type	Live:	Video:	Rationality
	% overimitation	% overimitation	Difference Ratings
Blocks	70	23	1.43 (1.97)
Duck	70	31	2.20 (1.75)
Elephant	65	19	1.94 (1.75)
Fan	23	15	1.71 (1.86)
Lion	72	31	2.14 (1.70)

Mean (and standard deviation) of rationality difference ratings for each apparatus type.

### Video vs Live Demonstration

**Figure 1:**
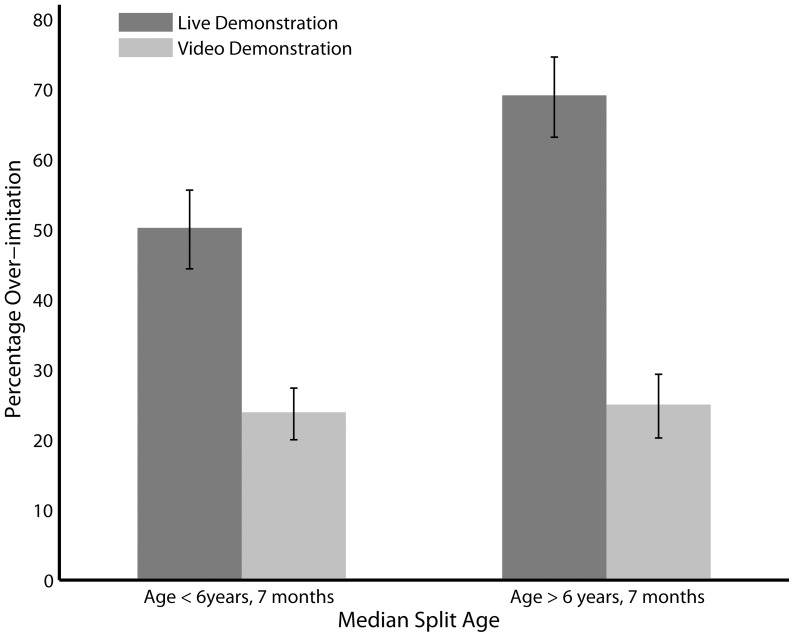
Overimitation score for younger and older children (based on a median split) as a function of demonstration type. The use of a median split for age is for visualisation purposes only; all analyses were run using age as a linear covariate.

Percentage overimitation for each age group as a function of demonstration type is presented in Figure 1. A significant main effect of demonstration type revealed that children were more likely to overimitate a model who demonstrated the action live, compared to a video demonstration (F(1,77) = 15.035, p<0.0001). There was also main effect of age (F(1,77) = 4.50, p<0.05), showing that older children were more likely to overimitate than younger children. No main effect of BPVS (F(1,77) = 0.09, p = 0.76) or SAS (F(1,77) = 0.14, p = 0.71) was found. Furthermore, when analysing a subset of the data in which groups were matched for BPVS and SAS (n = 39 in each condition), the same pattern of results was observed.

### Eye Contact Preceding Irrational Actions

**Figure 2:**
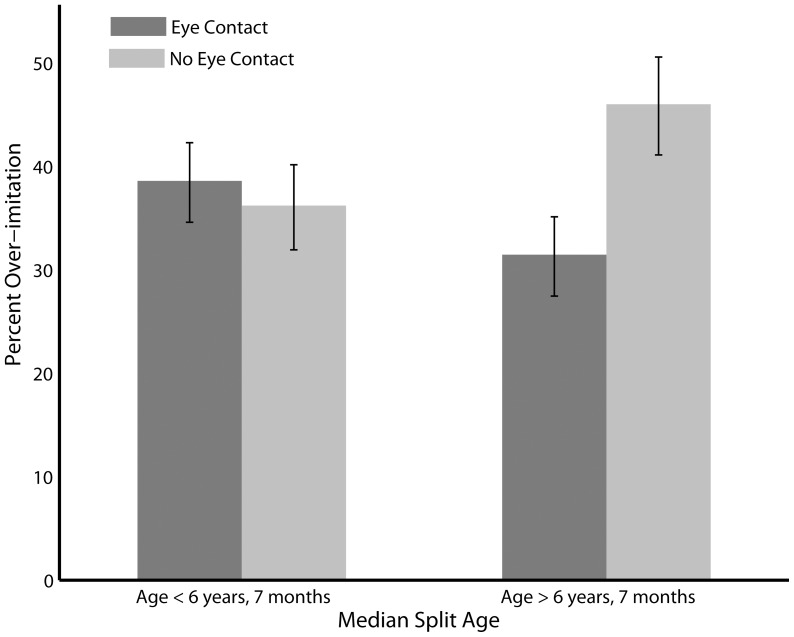
Overimitation score for younger and older children (based on a median split) as a function of preceding eye contact. The use of a median split is for visualisation purposes only; all analyses were run using age as a linear covariate.

Percentage overimitation for each age group as a function of preceding eye contact is presented in Figure 2. As with the previous analysis, a main effect of demonstration type (F(1,367) = 46.73, p<0.0001) and a main effect of age (F(1,367) = 7.05, p = 0.008) was present. No main effect of eye contact preceding an irrational action is reported (F(1,367) = 0.01, p = 0.97) although a significant age by eye contact interaction was found (F(1,367) = 5.99, p = 0.01). A post hoc t-test shows that this interaction is driven by an increase in overimitation in the older children when eye contact is absent (t(225) = 2.04, p = 0.04). In addition, an interaction between age and demonstration type (F(1,367) = 4.82, p = 0.03) was found. This suggests that as children get older, they are more likely to overimitate the live (t(214) = 2.48, p = 0.01) but not the video condition (t(263) = −0.26, p = 0.79).

### Rationality Ratings

**Figure 3:**
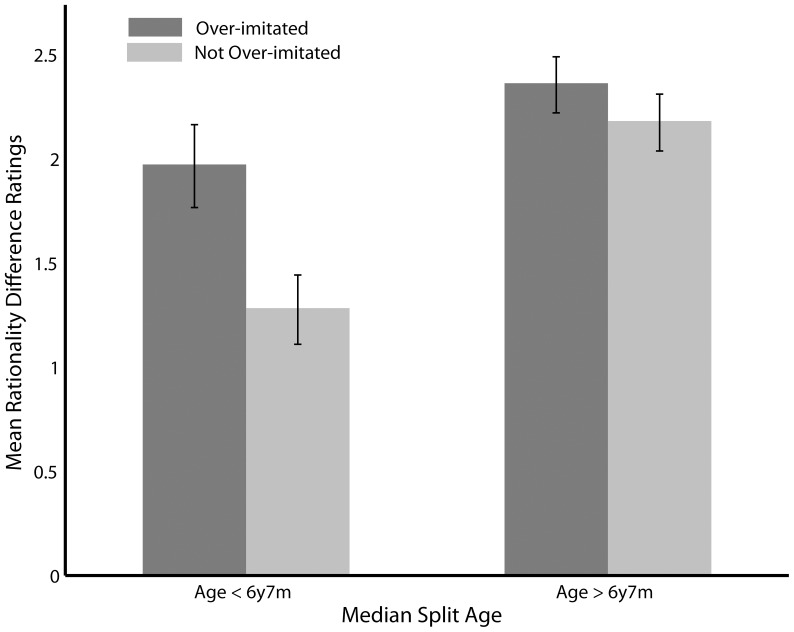
Mean difference in rationality ratings between rational and irrational actions that were either overimitated or not overimitated. Results are visualised using a median split for age but all analyses were run using age as a linear covariate.

Rationality difference ratings as a function of overimitation behaviour are presented in Figure 3. Children who overimitated an action subsequently rated that action as more irrational than the actions that they did not overimitate (F(1,364) = 3.89, p = 0.05). In addition, older children reported larger rationality differences between rational and irrational actions, compared to younger children (F(1,364) = 16.92, p<0.001). Effects of eye contact (F(1,364) = 0.31, p = 0.58), demonstration type (F(1,364) = 3.74, p = 0.06), BPVS (F(1,364) = 3.28, p = 0.07) and SAS (F(1,364) = 1.33, p = 0.25) were not significant.

### Predictors of overimitation behaviour

We used a logistic regression model to determine which of all the factors measured in this study best predicts overimitation behaviour. Results are shown in Table 4. The final model accounted for 26% of the variance in overimitation behaviour (NagelKerke R^2^ = 0.259) using four of ten variables. Firstly, overimitation was most likely when participants saw a live demonstration, compared to a video demonstration. Second, overimitation occurred less when participants were given the fan trial compared to all other trials. Rationality ratings predicted overimitation as the higher the rationality difference rating, the more likely the participant was to overimitate. Lastly, an age by eye contact interaction was also a significant predictor of overimitation, showing that in the older children, eye contact reduced propensity to overimitate. Child age, the age by rationality rating interaction, BPVS, SAS, demonstrator eye contact and the demonstration type by rationality rating interaction did not predict overimitation behaviour. Overall, this model was able to correctly predict overimitation behaviour on 73% of trials using these four variables.

**Table 4:** Factors entered into the binary logistic regression.

Variable	Beta	Wald	p
Demonstration Type	−1.41	34.38	0.0001
Apparatus Type (fan)	−1.70	16.28	0.0001
Rationality Ratings	0.14	4.08	0.04
Age x EC	0.48	9.49	0.002
Age	Excluded – step 1	-	-
Age x Rationality Ratings	Excluded – step 2	-	-
BVPS	Excluded – step 3	-	-
SAS	Excluded – step 4	-	-
Eye Contact	Excluded – step 5	-	-
Demonstration x EC	Excluded – step 6	-	-

## Discussion

The present study aimed to identify the social modulators of overimitation whilst reducing the demands of causal inference. We found substantial levels of overimitation which increased with age, despite testing older children than previous studies and using simple objects with minimal causal reasoning demands. The data also show that social cues had a larger impact on older children, who overimitated more following live demonstrations but less following eye contact. Finally, actions that were rated as least rational were more likely to be copied than the actions rated as more rational. We discuss what our results mean for social models of overimitation, causal reasoning models, and the development of overimitation in turn.

### Social models of overimitation

Based on previous studies [4], [13], we predicted that overimitation would increase with increases in social engagement, that is, with live demonstration and with eye contact. Our predictions were confirmed for the video compared to live demonstration comparison. Across all ages in our sample, the live demonstrator was copied with higher fidelity than the videoed demonstrator. This effect is most likely to be because the social presence is stronger in the live condition. This is consistent with previous findings which suggest that increased social engagement elicits higher levels of overimitation [4], [13]. Alternatively, a video deficit in imitation may also explain these findings [38]. It has been consistently demonstrated that infants show reduced imitation of video demonstrations compared to live demonstrations. This may be due to the video demonstration being degraded in quality, reduced in size, reduced from 3D to 2D or affording less relevance to the observer (see [39] for a review). However, the video deficit has not been examined in school-aged children and it is and is reported to be diminished by the age of 3-years [40]. Therefore, we believe that the most parsimonious explanation for the increase in overimitation for live demonstrations is the increased affordance for social interaction with the demonstrator in this condition (see [13] for a more detailed discussion of this issue).

In contrast, the eye contact manipulation did not yield the predicted results. Previous studies show that socially cued action sequences were overimitated more than uncued action sequences [4], and adults mimic faster following an eye contact cue [33]. If overimitation and mimicry are dependent on the same underlying mechanism [17], we would predict that direct eye contact prior to an unnecessary action should increase the propensity to imitate. Indeed, studies of social learning indicate that ostensive cues increase imitation fidelity [28], [41], [42]. However, the results from this manipulation were contingent upon the age of the participant. In the younger children there was no significant effect of eye contact on overimitation behaviour. In contrast, direct eye contact prior to an unnecessary action significantly reduced the propensity to overimitate in the older children.

This suggests we may need to re-think the role of eye contact during overimitation and what it may signal. One possibility is that as an ostensive cue, eye contact could be interpreted as a cue to ‘pay attention’ and think about the action performed. Previous studies have shown that ostensive cueing promotes gaze following [43] and directs attention towards the object being manipulated [28]. In the present study, eye contact might serve to make the children explicitly aware of the unnecessary action so that they choose not to copy it. This is likely to be particularly true for the simple objects used in this task as object learning is precluded. Another possibility is that eye contact may use up cognitive resources. Esseily and Fagard [28] report a reduction in imitation immediately following ostensive cueing in 10 month old infants. They argue that the social information is costly to process and cognitive resources may be depleted by ostensive cues. However, this explanation does not fit well with our data as the reduction in overimitation following eye contact is only present in the older children in our sample (6 years plus) who should have more cognitive resources than the younger children. A third possibility is that eye contact is interpreted as a cue to learn about the object and thus shifts the child's goal in the situation towards learning. According to Over and Carpenter [17], adopting a learning goal in an imitative task should reduce overimitation in the manner that we report. However, all of these are post-hoc explanations and future examination of the circumstances that alter a child's goal in an imitative situation would be valuable. In sum, the present results demonstrate that eye contact is a subtle cue that can be interpreted in different, context dependant ways. Further studies will be needed to understand the relationship between different eye contact cues in mimicry and those in overimitation.

### Causal reasoning models of overimitation

The present study used stimuli which are familiar to the child, with minimal causal reasoning demands. If causal misattributions are driving overimitation behaviour, we would expect to see very little overimitation in this task. Furthermore, we would expect overimitation behaviour to decrease with age and with rationality ratings. These predictions do not reflect the pattern of results that was observed. Sixty-two percent of children overimitated at least one trial in this sample, and rates of overimitation increased with age. Rationality ratings were collected to assess how children perceived each action. Somewhat surprisingly, the children who copied an unnecessary action subsequently rated it as more irrational than those who did not copy it. Again, this provides evidence against a causal learning explanation for overimitation behaviour as children who understand that an action is irrational (silly) are more likely to imitate that action. This finding adds to the existing literature as previously, ratings of actions have been taken prior to the child completing the actions, and thus potentially influencing subsequent imitation [15]. In addition, Kenward and colleagues [15] only included children who overimitated and as such, could not demonstrate the relationship between rationality ratings and behaviour that this study has identified. Previous studies that find evidence in support of the automatic causal encoding model have examined children under five years old [5], [9]. It is possible that overimitation in this young group is driven by causal reasoning, while social factors dominate in older children as causal reasoning and social skills develop.

### Developmental Changes in Overimitation

This study explored overimitation over the 5–8 year old age range. Like previous studies, we find that overimitation increases with age [30] in a way that is inconsistent with a causal reasoning explanation. Perhaps more interestingly we report two interactions between age and the social manipulations in this study (namely demonstration type and eye contact). In both of these interactions, sensitivity to the social components of the task increases with age. Previous studies that support automatic causal encoding hypothesis have tested younger children (2–5 year olds) and found persistence in overimitation despite social cues [5], [9]. Again, this data suggests that causal reasoning dominates responding in this younger age group, while social cues are much more important in the older children studied here. This should be investigated more thoroughly in a wider age range of children.

Individual difference measures of verbal intelligence and social ability did not predict overimitation. The lack of predictive power of social ability was surprising, considering that the social features of the task have a large influence on overimitation behaviour. However, this sample did not include a full range of social abilities and the SAS is a limited social measure. Studies examining overimitation behaviour in a sample of children with autism have yielded different results. Two previous studies have shown that children with autism faithfully imitate inefficient tool selection and use [23], [44] but overimitation was absent in participants with autism for visibly unnecessary actions with minimal causal demands [20]. These differences indicate that the apparatus types used have a huge bearing on social overimitation behaviour and further work should investigate precisely what object features are important for overimitation.

### Limitations

We would like to acknowledge two limitations of the current study. Firstly, due to the difficulty in developing appropriate stimuli and restriction on experiment length, the number of eye contact vs no eye contact trials was unbalanced within subject. The analysis of this data on trial-by-trial basis minimises the problems associated with unequal trial numbers and we believe the results to be unaffected by this. Secondly, the use of familiar objects in traditional imitation tasks is criticised [45] as participants can act on their prior knowledge of the object and therefore, it is difficult to distinguish imitative behaviour from normative behaviour. Contrary to this argument, results from our study show that despite object familiarity, children frequently complete unnecessary actions which are unlikely to have been produced without the demonstration of that action. We argue that in overimitation paradigms, the use of familiar objects actually strengthens our understanding of overimitation as causal reasoning explanations can be eliminated.

## Conclusions

The present study demonstrates that overimitation increases with age and is modulated by social cues, even in a task with minimal demands for causal reasoning. This argues against a pure causal learning explanation of overimitation, and demonstrates that social factors play a critical role in the decision about what to imitate. Older children showed greater sensitivity to social cues, demonstrating that development of social interaction skills continues over the primary school years.
